# Physical activity status and its association with quality of life among children with down syndrome in Saudi Arabia: A comparative cross-sectional study

**DOI:** 10.1371/journal.pone.0297111

**Published:** 2024-02-12

**Authors:** Amani S. Alqahtani, Maha F. Algabbani, Saad A. Alhammad, Khalid S. Alwadeai, Adel Alhusaini

**Affiliations:** Department of Health Rehabilitation Sciences, College of Applied Medical Sciences, King Saud University, Riyadh, Saudi Arabia; Universiti Malaya, MALAYSIA

## Abstract

**Background:**

Down syndrome is a genetic disorder that causes physical and cognitive challenges. Identifying the impact of sedentary behavior and physical activity on people with Down syndrome is crucial for early intervention. The purpose of this study is to compare physical activity and sedentary behavior among children with Down syndrome and typically developing children, as well as assess their relationship with quality of life.

**Methods:**

In the cross-sectional study, 67 children between the ages of 6 and 12 were enrolled: 29 in the Down syndrome group and 38 in the typically developing group. Each child wore an ActiGraph wGT3X-BT for seven days. Accelerometer data and quality of life data were analysed.

**Results:**

Physical activity and sedentary behavior were not significantly different between the Down syndrome and typically developing groups (*p* ˃ .05). With large effect sizes (partial eta squares ranging from 0.21 to 0.59), typically developing children had a significantly better quality of life than children with Down syndrome. There was a weak positive correlation between moderate physical activity and school performance in children with Down syndrome. For typically developing children, there is a weak negative correlation between light physical activity and physical function, school function, and total paediatric quality of life scale scores.

**Conclusions:**

This study indicates that children with Down syndrome have participated in more physical activities, resulting in a reduction in differences between them and typically developing children. Additionally, typically developing had higher quality of life than children with Down syndrome. For healthcare professionals and educators, these findings provide valuable insights into developing strategies to enhance physical activity for children with developmental disabilities.

## Introduction

Down syndrome is a genetic disorder characterized by the presence of an extra 21st chromosome, this additional chromosome leads to various physical and cognitive challenges [1]. One of the main physical challenges for children with Down syndrome is low muscle tone, also known as hypotonia, that can affect the strength and coordination of muscles, making it harder for these children to perform certain physical tasks [2]. As a result, functional activities are often difficult for them, resulting in a significant decline in their overall participation [3].

According to the guidelines provided by the World Health Organization (WHO), it is highly recommended that children between the ages of 5 and 17 engage in moderate to vigorous physical activity (MVPA) for at least 60 minutes each day [4]. Physical activity (PA) is "any bodily movement produced by skeletal muscles that requires energy expenditure" [4]. It classified into three categories based on energy expenditure: 1) Light Physical Activity (LPA), refers to activities with an energy expenditure of less than 3 Metabolic Equivalent of Task (METS), 2) Moderate Physical Activity (MPA), involves activities with an energy expenditure of 3 to less than 6 METS, and 3) Vigorous Physical Activity (VPA), refers to activities with an energy expenditure of 6 or larger METS. In contrast, sedentary behavior (SB) consists of any waking behavior characterized by a lower energy expenditure than 1.5 METS during sitting, reclining, or lying [4]. METS is a unit that quantifies physical intensity. Furthermore, researchers found that children should walk 12,000 steps a day according to various step-count guidelines [5]. This recommendation highlights the importance of regular movement in children’s daily routine.

Children with DS are often described as sedentary and less likely to engage in the recommended levels of physical activity compared to typically developing (TD) children. Previous studies found that children with DS had lower levels of physical activity compared to TD children of the same age [6–8]. Additionally, children with DS were not able to achieve the daily step count target [7].

Physical inactivity and sedentary lifestyles have negative effects on children with Down syndrome, lack of physical activity can lead to obesity, cardiovascular health issues, musculoskeletal problems, decreased cognitive function, and impact mental well-being [9]. Additionally, passive sedentary behavior is positively associated with depression symptoms [10]. In order to maintain good health and quality of life, it is important to promote and encourage regular physical activity [10–13].

In order to design and endorse successful interventions to maintain or increase physical activity levels, it is important to assess current levels of physical activity. While previous research has examined PA levels in DS children in Saudi Arabia, little is known about the relationship between PA and QoL. Therefore, the aims of this study were (1) to measure and compare the physical activity, SB time and step count objectively between children with DS and TD children; and (2) to examine the relationships of PA, SB time and step count with quality of life. The results of this study may indicate whether the level of physical activity has changed among children with DS over the most recent year in Saudi Arabia and whether children with or without DS meet the recommended guidelines regarding their PA level.

## Materials and methods

### Study design, sampling and setting

The study design was a quantitative descriptive comparative cross-sectional study. A letter of invitation was sent to the caregiver of each eligible child. Eighty participants, who agreed to participate, were recruited as a convenience sample: 40 children with Down syndrome (DS group) and 40 typically developing children (TD group). The children with DS were recruited from the Saudi Association for Special Education Center, Efada Center for Down Syndrome, National Center for Early Intervention and the rehabilitation centre in King Saud University Medical City located in Riyadh, Saudi Arabia. Children in the TD group were recruited from two different schools in the same region. The study was conducted from August to November 2021.

### Participants

The children with DS included in this study were (1) aged 6 to 12 years and (2) able to follow simple Arabic verbal instructions. If a child had a history of lower limb surgery, walked with a support device or had an illness or injury that could affect their physical function, they were excluded from the study. Similarly, TD children were included if they were (1) of the same age range as children with DS; aged 6 to 12 years and (2) able to follow simple verbal instructions in Arabic; they also had to have no medical issues or recent injuries. Both groups had male and female children. Children from both groups were excluded if they did not wear the accelerometers for the duration of the study.

Sample size estimated using G-Power Calculation Program (version 3.1.9.4), to find a medium effect size of 0.5 (based on Cohen’s d), a significance level of .05, and a power level of 0.80 with addition 10% add for the possibility of drop off, 80 participants were required (40 for each group).

### Ethical considerations and consent

The study was approved by the by the Institutional Review Board (IRB) in the Research Ethics Committee of the Medical College at King Saud University (No. 20/0673/IRB). Official permissions from the children’s schools were obtained. The parents signed a written formal consent form after receiving full information about the study before it began. The children provided assent as appropriate.

### Data collection

#### Sociodemographic and anthropometric characteristics

Sociodemographic data were reported by the caregivers. Each participant’s weight was measured to the nearest 0.1 kg while the child was in bare feet using an Eufy Body Sense Smart Scale [14]. Height was measured to the nearest 0.1 cm using a wall-mounted stadiometer and recorded. Body mass index (BMI) was calculated according to the following formula:

$$BMI=\frac{Weight\;(kg)}{Height\;squared(m^{2})}$$

BMI was converted to a percentile to define underweight (< 5th percentile), normal weight (5th to <85th percentile), overweight (85th to <95th percentile), and obese (≥95th percentile) for each participant [15].

#### Physical activity, sedentary behaviour and step count

A reliable and valid 3-axis ActiGraph wGT3X-BT accelerometer, for children with DS, was used to measure the frequency, duration and intensity of PA (LPA, MPA, VPA, and MVPA), as well as inactivity through low magnitude values and step count per day for seven consecutive days [16, 17]. The wGT3X-BT is a small, lightweight accelerometer that consists of three sensors that each measure acceleration in different planes. A wear time sensor on the back of the device detects when the wrist worn device has been removed automatically using capacitive touch technology [16].

The participants’ data, including height, weight, gender, non-dominant hand, and date of birth, were recorded using the ActiLife 6 Data Analysis Software, PA and SB times in minutes were calculated from the raw data obtained from the accelerometer, based on 60-second epoch length files. In addition, the step count was recorded and used as an indicator of PA level [16].

#### Quality of life measure

Paediatric quality of life scales (PedsQL) are valid and reliable measures of quality of life in children with Down syndrome [18]. The Arabic version of PedsQL, specifically, the parents’ proxy scale, was used to assess the parental perceptions of each child participant’s quality of life (QoL) [19]. An important aspect of PedsQL is that it covers the core dimensions of health as in outlined by the World Health Organization (physical, social, and emotional functioning), along with the school’s role health [19].

The core scales (domains) contain 23 items: eight items of physical function, five items of emotional function, five items of social function, and five items of school performance. The results were converted to a score range of 0–100 as follows: 0 = 100, 1 = 75, 2 = 50, 3 = 25, and 4 = 0. The score of each domain was calculated using the following formula:

$$Score=\frac{The\;sum\;of\;the\;items}{The\;number\;of\;items\;that\;were\;answered\;by\;participant}$$

Overall, the QoL score was calculated by summing all the items reported in all domains, where 100 represented the highest (or best) QoL. The scores were categorised into three groups: low (PedsQL < .50), moderate (.50 ≤ PedsQL < .80), and high (PedsQL ≥ .80) [19].

#### Procedures

During the first session, the sociodemographic, anthropometric and QoL data were collected. Participants were instructed to wear the accelerometer on the wrists of the nondominant hand for seven consecutive days and remove it during sleeping and water-based activities, such as swimming or bathing. Participants were provided with a diary sheet to record how long the child wore the accelerometer over the course of the seven days.

#### Statistical analysis

Analyses were conducted using Statistical Package for Social Studies (SPSS) Version 19 for Windows (IBM SPSS, Armonk, NY, USA). The distribution of the data was examined for normality using the Shapiro–Wilk test. The data are presented as means and standard deviations for normally distributed continuous data or medians and interquartile ranges (first and third quartiles) for non-normally distributed continuous data. Frequencies and percentages are used to describe data from categorical variables.

The differences in sociodemographic and anthropometric measures between the DS and TD groups were assessed using the chi-square test for categorical variables and the independent sample t-test (α = .05, 95% confidence interval) or Mann-Whitney U test (α = .05) according to the data distribution for the continuous variables.

A two-way multivariate analysis of covariance (MANCOVA) was performed to evaluate the effect of the group, gender and the interaction of group and gender on the variables PA, SB and step count, using age as a covariate. To compare the time spent on SB and different PA levels for each group, a repeated measures ANCOVA was conducted using age as a covariate. A two-way multivariate analysis of variance (MANOVA) was performed to evaluate the differences in QoL between the two groups and measure the effect size.

In the case of statistical significance, effect sizes were assessed using Cohen’s d (d < 0.3, small effect; 0.3 ≥ d ≤0.5, moderate effect; d ≥ 0.5, large effect) or partial eta square (pη^2^) (.01 to .06, small effect size; .06 to .14, medium effect size; and .14 or higher, large effect size). The effect size measure was determined according to the statistical test used [20].

Pearson’s or Spearman’s correlation coefficients were used to assess the relationships of PA and SB with QoL. At a .01 level of significance (99% confidence), the correlation values were interpreted as follows: 0–.19, very weak; .20–.39. weak; .40–.59, moderate; .60–.79, strong; and .80–1, very strong correlation [21]. At 95% confidence interval, all results were considered statistically significant at a *p*-value of < .05.

## Results

Out of the 80 participants recruited for this study, 13 children (11 children with DS and 2 TD children) were excluded because they did not wear the accelerometers for the complete duration of the study. Thus, 67 participants were included in the study data analysis; however, PedsQL scores were missing for two participants with DS, so they were excluded from the analysis of QoL. Shapiro–Wilk tests revealed that data were normally distributed for PA, SB and step count (*p* ˃ .05). However, the sociodemographic, anthropometric and PedsQL scores were not normally distributed (*p* < .05).

### Participants’ sociodemographic and anthropometric characteristics

The characteristics of the children and their caregivers are shown in Table 1. There were no significant differences in the sociodemographic and anthropometric variables between the DS and TD groups except for height; TD children were significantly taller than children with DS. None of the children with DS were underweight. The percentage of children with obesity was higher in children with DS (27%) than in TD children (15%). Almost all the children (66, 98.51%) from both groups were enrolled in schools. All the children were accompanied by their parents.

**Table 1:** Participant characteristics.

Variable		DS group	TD group	*p* value
		N = 29	N = 38	
**Child characteristics**				
Age (years)		9.67(8.58, 11,17)	10.13(7.81,11,52)	.81^a^
Gender	Boys	17 (58.6)	13 (34.2)	.08^b^
	Girls	12 (41.4)	25 (65.8)	
Height in cm		125 (108, 132)	135(121, 146.25)	.01^a^ *
Weight in kg		30 (20, 41.5)	28 (23, 40.75)	.85^a^
Body mass index in kg/m^2^		19.1(16.4, 22.3)	17.55(14.80, 21.08)	.10 ^a^
Body percental category N (%)	Underweight	0	7 (18.4)	.09 ^b^
	Healthy	14 (48.3)	16 (42.1)	
	Overweight	7 (24.1)	9 (23.7)	
	Obese	8 (27.6).	6 (15.2)	
**Caregiver characteristics**				
Mother’s education	High school or less	12	10	.19 ^b^
	Bachelor or higher	17	28	
Father’s education	High school or less	6	12	.23 ^b^
	Bachelor or higher	23	26	
Income per month	<9,000 SR	7	16	.23 ^b^
	9,000 to 12,000 SR	6	4	
	> 12,000 SR	16	18	
Type of house	single-family home	24	33	.64 ^b^
	multi-family home	5	5	

N = number of participants, DS = Down syndrome, TD = Typically developing, cm = centimetre, kg = kilogram

Continuous data were represented by median (1st, 3rd quartiles), and categorical data were represented by frequency and percentage.

a = Mann–Whitney U test

b = chi-square test

* Significant at level *p* < .05

### Physical activity, sedentary behaviour, and step count

Descriptive data for PA, SB and step count are shown in Table 2. A total of 25 (86.2%) children with DS and 33 (86.8%) TD children exceeded the step count per day recommended by the guidelines. However, none of them reported any VPA, which implied that none of the children met the recommended PA level.

**Table 2:** Descriptive statistics for PA variables.

Variables	DS group			TD group			Total	
	Girls	Boys	Total	Girls	Boys	Total	Girls	Boys
	N = 12	N = 17	N = 29	N = 25	N = 13	N = 38	N = 37	N = 30
SB (min/day)	614±226	646±106	633±163	694± 120	657±133	682±124	668±163	651±117
LPA (min/day)	468±111	470±108	469±107	469.±82	497±95	479±86	469±90	481±102
MPA (min/day)	152±97	195±81	177±89	194±73	165±85	184±77	180±82	182±83
Step count per day	17,360	19,209	18,444	17,965	18,404	18,115	17,769	18,860
	±5,470	±6,386	±5,993	±4,807	±5,760	±5,079	±4,963	±6,033

Data presented as mean ± standard deviation. SB: sedentary behaviour; LPA: light physical activity; MPA: moderate physical activity; DS: children with Down syndrome; TD: typically developing children; N = number of participants.

The MANCOVA analysis revealed that there were no main effect of group, gender, and the interaction of group and gender on PA, SB and step count (see Table 3). Thus, there were no significant differences between children with DS and TD children or significant differences between boys and girls (*P* > .05).

**Table 3:** Two-way MANCOVA for the main effect of group and gender and the interaction of group and gender on physical activity, sedentary behaviour and step count.

Source	Dependent variable	df	F	Sig.	pη2	95% CI for difference	
						Lower bound	Upper bound
Group	SB	1, 62	1.86	.18	.029	-831	156
	LPA	1, 62	0.28	.60	.004	-434	252
	MPA	1, 62	0.03	.87	.000	-278	237
	Step count per day	1, 62	0.03	.85	.001	-2398	2886
Gender	SB	1, 62	0.19	.66	.003	-618	393
	LPA	1, 62	0.07	.79	.001	-397	305
	MPA	1, 62	0.33	.57	.005	-188	339
	Step count per day	1, 62	0.03	.87	.000	-2474	2935
Group * Gender	SB	1, 62	0.99	.32	.016	-	-
	LPA	1, 62	0.28	.60	.004	-	-
	MPA	1, 62	3.73	.06	.057	-	-
	Step count per day	1, 62	0.27	.60	.004	-	-

SB: sedentary behaviour; LPA: light physical activity; MPA: moderate physical activity; df: degrees of freedom, F: F-value, Sig.: significance level (< .05); pη^2^: partial eta-squared; CI: confidence interval.

We found a significant difference between the time spent on sedentary activity, light PA, and moderate PA for the DS group (F_1,27_ = 5.81, *p* = .02) and also for the TD group (F_1,36_ = 6.89, *p* = .01). Both groups spent most of their time on SB; less time was spent on moderate PA.

### Quality of life measure

The total QoL scores and the scores of each domain were significantly higher for the TD children than for the children with DS; the effect size was large and the statistical power achieved ranged between .66 and .88 (see Table 4). Children with TD had higher QoL scores (>80), while those with DS had moderate QoL scores (between 50 and 80).

**Table 4:** Two-way MANOVA analysis of the QoL differences between children with DS and TD children.

PedsQoL domain	DS group		TD group		df, Error	F	Sig.	pη^2^	95% CI for difference		Power achieved
	N = 27		N = 38								
	Mean±SD	Median (1^st^, 3^rd^)	Mean±SD	Median (1^st^, 3^rd^)					Lower bound	Upper bound	
Physical functioning	73.3±14.1	71.8 (65.6, 84.3)	93.5±8.6	96.8 (90.6,100.0)	1, 63	50.7	< .001	.45	-25.77	-14.48	.81
Emotional functioning	74.1±12.9	75.0 (65.0, 80.0)	92.4±11.4	97.5 (85.00,100.0)	1, 63	36.4	< .001	.37	-24.35	-12.24	.78
Social functioning	71.9±19.1	70.0 (55.0, 90.0)	98.0 ± 3.9	100.0 (98.7, 100.0)	1, 63	67.9	< .001	.52	-32.52	-19.83	.85
School functioning	75.2±19.9	80.0 (70.0, 80.0)	91.6±12.7	95.0 (88.7, 100.0)	1, 63	16.5	< .001	.21	-20.07	-7.16	.66
Total	73.6±9.6	70.6 (67.4, 81.5)	93.8±7.6	95.6 (91.3, 100.0)	1, 63	90.5	< .001	.59	-24.48	-15.98	.88

df: degrees of freedom, F: F-value, Sig.: significant level (< .05); pη^2^: partial eta-squared. SD: standard deviation; N: number of participants; DS: Down syndrome; TD: typically developed; CI: confidence interval.

### Correlation analysis

For children with DS, there was a significant weak positive correlation of MPA per day with school function. The school function QoL level increased with an increase in the time spent on MPA. For TD children, there were significant negative correlations of LPA with physical function, school function and total PedsQoL scores. The total QoL (and the above-mentioned domains) decreased with more time spent on LPA (see Table 5).

**Table 5:** Correlations between PedsQoL and physical activity, sedentary behaviour and step count for both groups.

Group		Physical function	Emotional function	Social function	School function	Total PedsQoL
DS	SB	.249	.273	-.042	-.340	.003
	LPA	.064	-.017	-.239	.085	-.150
	MPA	-.198	-.334	.210	.384*	.066
	Step count per day	.016	-.153	.099	.316	.169
TD	SB	-.086	.003	-.143	-.208	-.092
	LPA	-.345*	-.381*	-.161	-.277	-.371*
	MPA	.169	.029	-.014	.202	.139
	Step count per day	-.067	-.141	-.046	-.037	-.110

DS: children with Down syndrome; TD: typically developing children; MET: Metabolic equivalent of task; SB: sedentary behaviour; LPA: light physical activity; MPA: moderate physical activity. *Spearman’s rho correlation was significant at a .05 level (2-tailed).

## Discussion

This study revealed that although both the children with and without Down syndrome did not meet the recommended physical activity level and most of their time was spent on SB, the majority exceeded the recommended step count per day. No significant differences appeared between the two groups or between different genders regarding time spent in SB, different PA level or step count per day. There was a significant difference in QoL between the children in favour of the TD participants. There was a weak correlation between school function and moderate PA for children with DS. For TD children, there was a positive weak correlation between light PA and physical function, social function and overall QoL. In addition, the children with DS in the sample were significantly shorter than TD children with a higher percentage of obesity.

These findings were consistent with the results of previous studies suggested that both children with and without Down syndrome often fail to meet the recommended levels of physical activity. Instead, they tend to engage in sedentary behaviors [6–8] this can be attributed to a combination of factors, such as lack of self-esteem [22], lack of accessibility to appropriate resources and environments [23], and the absence of adequate social support from parents, caregivers, and educators [24]. Moreover, the rise of technology, particularly screen-based devices, has contributed to the sedentary behavior observed in children with and without Down syndrome [25].

As suggested by previous study, achieving 60 minutes of MVPA per day is challenging for children with disabilities [6]. Our findings confirm those of Yamanaka et al., [6] in which children with DS spent more time in LPAs and MPAs than in VPAs. Several factors may contribute to this, including physical and cognitive manifestations, or safety confederations [1, 26].

In the current study, 86.20% of the children with DS achieved the recommended steps count per day, though, these steps were mostly spent in light PA rather than MVPA. The PA level, indicated by the step count, was higher compared to a previous study conducted with a similar population [7]. According to Alhusaini et al., [7] none of the children with DS reached the recommended step count measured by a pedometer. This could indicate that the physical activity of children with DS has improved during the past few years. Although we cannot confirm that since the different of assessment tool have been used.

Many studies have concluded that children with intellectual disabilities, including DS, are less physically active and more sedentary than TD children [27–29]. In contrast to previous studies, there were no significant differences between the groups in the current research. All children spent more time in SB than in PA, without significant differences. Therefore, the current study suggested that children with DS are not less likely to engage in regular PA than TD children. This is described in previous studies. Diaz [30] showed similar results; though, a questionnaire was used to capture the physical activity level instead of an objective measure, such as an accelerometer. The absence of differences may also indicate efforts made by those in care centers for children with DS to promote physical activity among these children. From another perspective, the observed absence of differences between the two groups in PA level could also be attributed to a general absence of PA in children. This is regardless of whether they are disabled [31].

In spite of PA’s promotion, a knowledge gap remains regarding the correlation between PA and QoL among children with DS. The study aimed to fill that knowledge gap. It revealed that children with DS reported a moderate QoL in line with the study by Rojnueangnit et al [32]. In addition, TD children had a better QoL than had been reported in previous research [32]. To the authors’ knowledge, there has been no study conducted in Saudi Arabia to measure the correlation between QoL and PA for children with DS. However, previous studies found that a higher frequency of physical activity was related to a better QoL in children with or without disabilities [11]. This study’s results were consistent with their findings. Physical activity may contribute to an improved QoL by improving physical health, mental well-being, social interaction, sleep quality, and energy levels.

Another interesting finding was that there was no effect of gender on PA; the PA of girls and boys was similar. This may indicate that the prospects of girls participating in physical activity have changed [33]. In recent years, there has been a concerted effort to empower a greater number of women and inspire them to lead healthier lives through physical fitness and active lifestyles. As a result of this dedicated efforts, there has been a notable increase in the participation of girls in sports activities [34–36].

The strength of this study is that it provides valuable information regarding PA levels based on objectively measured PA and their relationship to QoL in children with and without DS. A limitation of this study was the small sample size, considering the number of children who were unable to wear the accelerometer for the entire study period. More research with a larger randomised sample cover should be conducted to be able to better generalise the results and to examine the influence of health and cultural background on PA in children with different ethnic backgrounds. Furthermore, ActiGraph’s wGT3X-BT accelerometer does not have the capability to measure water-based activities. In spite of its reliability for tracking physical activity, it has limitations when it comes to water-based activities, so accurate measurements must be sought elsewhere.

## Conclusion

This study provides valuable insights into PA changes among children with and without DS. The findings indicate that the level of PA among children with DS has increased, leading to a reduction in differences between children with DS and TD children. Additionally, the study found no significant effect of gender on PA levels. Moreover, QoL was higher for TD children than children with DS. These findings contribute to the growing literature on PA in children with DS. They provide valuable insights for healthcare professionals and educators in developing strategies to promote physical activity and enhance the quality of life for children with DS. In order to gain a comprehensive understanding of physical activity patterns among children, longitudinal studies are recommended.

Through the creation of a supportive and inclusive environment, we recommend that physical activity be promoted and encouraged for children with or without Down syndrome. By engaging in regular physical activity, you can lead a healthy and active life. In addition to enhancing physical fitness, it also enhances cognitive, emotional, and social development.

## Supporting information

S1 Checklist STROBE statement—checklist of items that should be included in reports of *cross-sectional studies*. (DOCX)

S1 Data (XLSX)
